# Psychological Effects of the COVID-19 Imposed Lockdown on Adults with Attention Deficit/Hyperactivity Disorder: Cross-Sectional Survey Study

**DOI:** 10.2196/24430

**Published:** 2020-12-15

**Authors:** Marios Adamou, Tim Fullen, Nazmeen Galab, Isobel Mackintosh, Karl Abbott, Deborah Lowe, Claire Smith

**Affiliations:** 1 School of Human and Health Sciences University of Huddersfield Huddersfield United Kingdom; 2 South West Yorkshrire Partnership NHS Foundation Trust Wakefield United Kingdom

**Keywords:** adult ADHD, pandemic, lockdown, COVID-19, well-being, psychological, intervention, ADHD

## Abstract

**Background:**

The psychological effects of the COVID-19 government-imposed lockdown have been studied in several populations. These effects however have not been studied in adult populations with attention deficit/hyperactivity disorder (ADHD).

**Objective:**

We wanted to investigate the psychological effects of the COVID-19 imposed lockdown on an adult population with ADHD.

**Methods:**

We conducted a cross-sectional survey by administering the Patient Health Questionnaire-9, Generalized Anxiety Disorder Scale-7, Brief Adjustment Scale-6, Perceived Stress Scale, and Multidimensional Scale of Perceived Social Support to a pragmatic sample of adults with ADHD.

**Results:**

In total, 24 individuals (male: n=18, 75%; female: n=6, 25%; age: mean 21.75 years, SD 1.85 years) were included in this study. The adults with ADHD we surveyed had significant levels of emotional distress during the COVID-19 pandemic period. However, there was no evidence of significant deterioration to the mental health of our sample during the COVID-19 pandemic.

**Conclusions:**

When treatment for ADHD is maintained, the effects of the COVID-19 pandemic on the mental health of adults with ADHD are mild. Targeted psychological interventions may be useful in such circumstances.

## Introduction

Attention-deficit/hyperactivity disorder (ADHD) is one of the most common neuropsychiatric conditions. The pooled worldwide prevalence of ADHD is estimated to be approximately 5% in school-aged children, and impairing symptoms have persisted in adulthood in up to 65% of cases. The pooled estimated prevalence of ADHD in adults is approximately 2.5% [1]. ADHD is characterized by a persistent and impairing pattern of inattention and hyperactivity/impulsivity that causes significant impairment across many domains of mental health [2]. Along with these 3 main symptoms, people with ADHD also present with deficits in executive functions, behavior and emotion regulation, and motivation [3].

In December 2019, an outbreak of a novel coronavirus pneumonia occurred in Wuhan (Hubei, China), and subsequently attracted worldwide attention [4]. As a result, countries around the world introduced public health policies that have been commonly called a lockdown. These policies, which include individuals isolating at home and only allowing necessary movement, were indented to reduce the spread of the virus. The United Kingdom was put into lockdown on March 23, 2020.

As the lockdown progressed, concerns were raised about the effect of this lockdown on the mental health of individuals [5]. A general population survey administered by Ipsos MORI [6] has revealed widespread concerns about the impact of social isolation and social distancing on a person’s well-being, such as increased anxiety, depression, stress, and other negative feelings.

The mechanism behind the impact of the lockdown can be understood by considering the lockdown as an event that has created stressful life events in a person’s life. Stressful life events are described as discrete, quantifiable circumstances that can have a severe negative impact. A wealth of published evidence has shown that life event stressors are associated with the onset of depressive episodes and that there is a dose-response relationship between these stressors and depressive episodes [7]. Stressors are associated with severe initial depressive symptoms in both adult patients [8] in nonpatient community samples [9].

Stress is generally conceptualized as a negative cognitive-emotional state caused by an individual’s perception of difficulty in adjusting to or managing life events [10]. Studies have shown that adults with ADHD have elevated physiological stress responses and higher levels of self-reported subjective stress compared to nondiagnosed controls [11,12]. In fact, the levels of stress for adults diagnosed with ADHD appear elevated in the presence of a stressor and in anticipation of the stressor. In addition, a study has suggested that adults with ADHD have greater difficulty in recovering from elevated stress levels than adults without ADHD [12], indicating that persons with ADHD may also face challenges in recovering from stress.

Based on this context, we believe that adults with ADHD are disproportionately affected by the lockdown compared to other populations. We therefore administered a survey to a group of adults with ADHD to investigate the psychological effects of the COVID-19 imposed lockdown on this population, understand this population’s needs, and provide appropriate support.

## Methods

We conducted a cross-sectional survey study. The convenience sample was derived from the caseload of the ADHD and Autism Service at South West Yorkshire Partnership NHS Foundation Trust. The ADHD and Autism Service provides input to approximately 1.2 million people in West Yorkshire, England, for people with ADHD and autism. The ADHD and Autism Service can only offer pharmacological interventions to people with ADHD aged >25 years. Therefore, only people aged <25 years were eligible for this study. Our survey was considered a service improvement activity because the results of the survey were to be used to identify people who were suitable for COVID-19–specific psychological intervention.

Our cohort consisted of 94 individuals with a preestablished diagnosis of ADHD (male: n=75; female: n=19) who were first contacted on May 18, 2020, for a period of 2 weeks over the telephone. After 3 unsuccessful attempts of making contact with an individual, the person was considered not-responding, and no further attempts were made. All individuals were receiving pharmacological treatment.

For the people who did respond to the telephone call, after the purpose of the well-being survey was explained using a predetermined script, the Patient Health Questionnaire-9 (PHQ-9) [13], Generalized Anxiety Disorder Scale-7 (GAD-7) [14], Brief Adjustment Scale-6 (BASE-6) [15], Perceived Stress Scale (PSS) [16], and Multidimensional Scale of Perceived Social Support (MSPSS) [17] were administered.

The PHQ-9 and GAD-7 use a scale of 0 (not at all) to 3 (nearly every day) to measure the severity of self-reported depression and anxiety, respectively, within the last two weeks. They have 9 and 7 questions, respectively. PHQ-9 scores ranging from 0 to 5 represent mild depression, 6-10 represent moderate depression, 11-15 represent moderately severe depression, and 16-20 represent severe depression. GAD-7 scores of ≤4 predict no anxiety, 5-9 predict mild anxiety, 10-14 predict moderate anxiety, and 15-21 predict severe anxiety. 

The BASE-6 measures a person’s general psychological adjustment within the last week, with ratings between 1 (not at all) and 7 (extremely) given for each of the 6 questions.

The PSS consists of 10 items designed to assess the degree to which common life situations in the past month are deemed stressful, and responses are made using a 5-point scale (0=never, 4=very often). Individual scores on the PSS can range from 0 to 40, with higher scores indicating higher perceived stress. Scores ranging from 0 to 13 indicate low perceived stress, 14-26 indicate moderate perceived stress, and 27-40 indicate high perceived stress.

The MSPSS measures the perception of support from the following 3 sources: family, friends, and a significant other. The scale is comprised of 12 items on a 7-point Likert scale, with 4 items for each subscale. Higher scores reflect more perceived social support. Mean scores ranging from 1 to 2.9 indicate low perceived support, 3-5 indicate moderate perceived support, and 5.1-7 indicate high perceived support.

The results from these scales, along with demographic information, were recorded using Microsoft Excel. The results were then exported to IBM SPSS statistical software for analysis.

## Results

Of the 94 individuals eligible for the survey, 75 were male and 19 were female. The mean age of the total sample was 22.05 years (SD 1.837 years). Of these 94 individuals, 70 (74%; male: n=57; female: n=13) were excluded from this study, either because they could not be contacted or because they refused to take part in this study. Of these 70, 18 (26%; male: n=16; female: n=2) declined to take part in this study and 52 (74%; male: n=41; female: n=11) could not be contacted. In total, 24 patients (male: n=18, 75%; female: n=6, 25%) were included in this study. 

There was no significant difference in age between males who were excluded from this study (mean 22.26 years, SD 1.86 years) and included males (mean 21.94 years, SD 1.74 years; *t_73_*=0.64; *P*=.53). There was also no significant difference in age between females who were excluded from this study (mean 21.62 years, SD 2.18 years) and included females (mean 21.17 years, SD 1.83 years; *t_17_*=0.47; *P*=.65).

For the 24 included participants, there were no missing data. The scores from the scales were checked for normality using the Kolmogorov-Smirnov test. All scores had a normal distribution. The mean age of all included patients was 21.75 years (SD 1.85 years). Based on our survey results, the mean PSS score was 21.5 (SD 7), the mean MSPSS score was 5.55 (SD 0.89), the mean GAD-7 score was 4.84 (SD 1.46), the mean PHQ-9 score was 10.88 (SD 6.83), and the mean BASE-6 score was 17.13 (SD 9.56).

**Figure 1:**
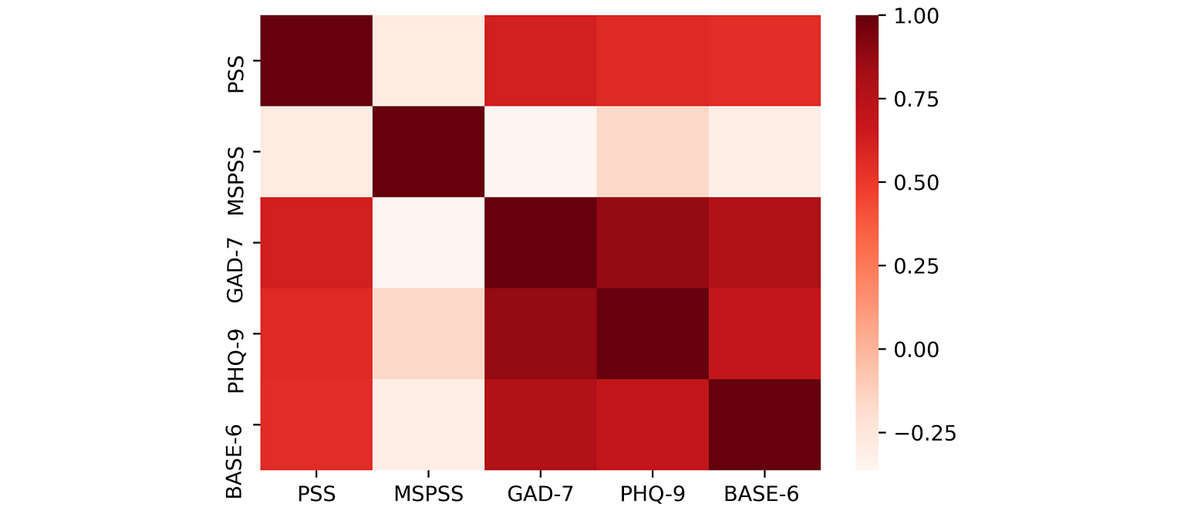
Heat map of correlations between the survey scale scores. BASE-6: Brief Adjustment Scale-6; GAD-7: Generalized Anxiety Disorder Scale-7; MSPSS: Multidimensional Scale of Perceived Social Support; PHQ-9: Patient Health Questionnaire; PSS: Perceived Stress Scale.

A Pearson correlation coefficient was computed to assess the relationship between the variables. We found a positive correlation between PSS scores and GAD-7 (*r*=.623; *P*=.01), PHQ-9 (*r*=.572; *P*=.03), and BASE-6 (*r*=.553; *P*=.05) scores; GAD-7 scores and PSS (*r*=.623; *P*=.01), PHQ-9 (*r*=.872; *P*<.001) BASE-6 (*r*=.778; *P*<.001) scores; and PHQ-9 scores and PSS (*r*=.572; *P*=.03), GAD-7 (*r*=.872; *P*<.001), BASE-6 (*r*=.696; *P*<.001) scores. All correlations were significant at the .01 level and all tests were 2-tailed. There was no correlation between age or MSPSS scores with any other variables (Figure 1)**.**

## Discussion

### Principle Results

Our study showed that the adults with ADHD we surveyed had significant levels of emotional distress during the COVID-19 pandemic. It was not clear if these levels were due to the psychological effects of the pandemic and the social changes it imposed or just what would be normally expected in this population. Furthermore, the pragmatic study design did not allow us to attribute a differential negative impact to the effect of the pandemic on this population and the general population. However, a discussion of the wider context of our findings generated some interesting ideas for future research.

In terms of anxiety, it is already known that adults with ADHD are associated with positive screening results [18]. In a sample of 141 adults with ADHD, Goniu and Moreno [19] reported that 48 (34.1%) adults met the criteria for mild anxiety, 31 (22%) met the criteria for moderate anxiety, and 18 (6.7%) met the criteria for severe anxiety. For our sample, the GAD-7 scores were in the mild to moderate range, even in the context of the global pandemic, indicating that our participants’ anxiety was not much worse than what has been previously reported.

In terms of depression, surprisingly, there was not much information in the current literature to know what the expected PHQ-9 scores would be for adults with ADHD. We know that the expected prevalence rate of PHQ-9 depressive disorders in the general population is 9.2% [20] and that the PHQ-9 scores we reported (mean 10.88, SD 6.83) reliably differentiate our study population from the general population. However, we cannot claim that this difference was a result of the impact of the pandemic, especially when the comorbidity rate for adult ADHD with major depressive disorder has been reported to be as high as 16% [21], depending on the sample.

We used the PSS to measure participants’ perception of stress. We believed that there was adequate reason for any person to be under some sort of personal stress due to the lockdown and its consequences for social life. For adults with ADHD specifically, it has been argued that their perception of stress would be even higher, as it has been suggested that adults with ADHD experience elevated perceived or subjective stress compared to adults without ADHD [22]. Our participants’ perceived stress was only slightly higher than those reported in adults without ADHD [23]. However, the standard deviation in our sample’s PSS scores suggest greater variability, which may be attributable to the social consequences of the lockdown. There is not enough research to allow us to make accurate comparisons with this scale for adults with ADHD. However, another study that used the PSS recruited a sample of people with posttraumatic stress disorder [24], and another study on people with ADHD [21] reported a lower mean PSS score than our study (mean 16.7, SD 1.6).

In terms of the level of psychological adjustment in 1 week, before we contacted our sample, their mean BASE-6 score was 17.13 (SD 9.56), which was similar to the clinical sample (mean 18.9, SD 7.0) and college student sample (mean 18.1 SD 6.8) scores reported during the evaluation of the BASE-6 [15]. However, it should be noted that the clinical and college student sample scores were reported outside of a pandemic context. Therefore, there is no evidence to suggest that the level of psychological adjustment of our sample was impaired when measured using the BASE-6. Furthermore, the reason why our participants’ BASE-6 scores were not as high as expected was not because social support was available. This is because participants’ MSPSS scores did not correlate with their BASE-6 scores.

Our participants’ mean MSPSS score was 5.55 (SD 0.89), which indicated a high level of support. Previous studies that used this scale for people with ADHD [25,26] have reported that the perception on the importance of social support between people with ADHD and people without ADHD was similar, even though the scores differed between the groups.

In this study, the GAD-7, PHQ-9, PSS, and BASE-6 scores positively correlated with each other, suggesting that in circumstances where the impact of stress is poor, psychological adjustment, mood, and anxiety will deteriorate. Based on this, it might be useful to have an available form of psychological intervention specific to current circumstances to prevent further deterioration.

We are aware that this study has several limitations; the sample size was small, the dropout rate was high, and the sample was not representative of all adults due to only including people aged 18-25 years. Despite this, our findings provided a window into how adults with ADHD fared with the social changes caused by a global pandemic that restricted their movement and relationships. In our sample, participants seemed to manage the same as their expected baseline, and this may be somewhat explained by the fact that they were able to continue their medication. Our findings also suggest that it may be useful to have an available form of psychological intervention for increasing resilience in adults with ADHD, in case other life events or a prolonged pandemic cause more severe deterioration to their mental health.

### Conclusions

We expected that adults with ADHD would find the restrictions imposed by the lockdown particularly difficult to adhere to, and as a result, their mental health would be at risk of deterioration. Our findings suggest that there was evidence of mental ill health, but this was not severe or disproportionate to what was expected for this population. This may have been due to all participants continuing their pharmacological treatment for ADHD. However, due to their reported distress, a preventative targeted psychological intervention may be useful.

## Abbreviations

ADHD: attention deficit/hyperactivity disorder
BASE-6: Brief Adjustment Scale-6
GAD-7: Generalized Anxiety Disorder Scale-7
MSPSS: Multidimensional Scale of Perceived Social Support
PHQ-9: Patient Health Questionnaire
PSS: Perceived Stress Scale
